# Replication Inhibition of Hepatitis B Virus and Hepatitis C Virus in Co-Infected Patients in Chinese Population

**DOI:** 10.1371/journal.pone.0139015

**Published:** 2015-09-30

**Authors:** Ge Yu, Xiumei Chi, Ruihong Wu, Xiaomei Wang, Xiuzhu Gao, Fei Kong, Xiangwei Feng, Yuanda Gao, Xinxing Huang, Jinglan Jin, Yue Qi, Zhengkun Tu, Bing Sun, Jin Zhong, Yu Pan, Junqi Niu

**Affiliations:** 1 Department of Hepatology, The First Hospital of Jilin University, Changchun, Jilin Province, China; 2 Key Laboratory of Zoonosis Research, Ministry Education, Jilin University, Changchun, Jilin, China; Jilin Province Key Laboratory of Infectious Diseases, Laboratory of Molecular Virology, Changchun, Jilin Province, China; 3 Institute Pasteur of Shanghai, Shanghai Institutes for Biological Sciences, Chinese Academy of Sciences, Shanghai, China; Centers for Disease Control and Prevention, UNITED STATES

## Abstract

**Background:**

Hepatitis B virus (HBV) and hepatitis C virus (HCV) co-infections contributes to a substantial proportion of liver disease worldwide. The aim of this study was to assess the clinical and virological features of HBV-HCV co-infection.

**Methods:**

Demographic data were collected for 3238 high-risk people from an HCV-endemic region in China. Laboratory tests included HCV antibody and HBV serological markers, liver function tests, and routine blood analysis. Anti-HCV positive samples were analyzed for HCV RNA levels and subgenotypes. HBsAg-positive samples were tested for HBV DNA.

**Results:**

A total of 1468 patients had chronic HCV and/or HBV infections. Among them, 1200 individuals were classified as HCV mono-infected, 161 were classified as HBV mono-infected, and 107 were classified as co-infected. The HBV-HCV co-infected patients not only had a lower HBV DNA positive rate compared to HBV mono-infected patients (84.1% versus 94.4%, respectively; P<0.001). The median HCV RNA levels in HBV-HCV co-infected patients were significantly lower than those in the HCV mono-infected patients (1.18[Interquartile range (IQR) 0–5.57] versus 5.87[IQR, 3.54–6.71] Log_10_ IU/mL, respectively; P<0.001). Furthermore, co-infected patients were less likely to have detectable HCV RNA levels than HCV mono-infected patients (23.4% versus 56.5%, respectively; P<0.001). Those HBV-HCV co-infected patients had significantly lower median HBV DNA levels than those mono-infected with HBV (1.97[IQR, 1.3–3.43] versus 3.06[IQR, 2–4.28] Log_10_ IU/mL, respectively; P<0.001). The HBV-HCV co-infection group had higher ALT, AST, ALP, GGT, APRI and FIB-4 levels, but lower ALB and total platelet compared to the HBV mono-infection group, and similar to that of the HCV mono-infected group.

**Conclusion:**

These results suggest that co-infection with HCV and HBV inhibits the replication of both viruses. The serologic results of HBV-HCV co-infection in patients suggests more liver injury compared to HBV mono-infected patients, but is similar to HCV mono-infection.

## Introduction

Hepatitis B virus (HBV) and hepatitis C virus (HCV) infections are the most common causes of liver disease worldwide. An estimated 350 million individuals have chronic HBV infection, and 170 million individuals have chronic HCV infection [1–3]. HBV and HCV have similar modes of transmission [4], and co-infections occurs frequently in endemic areas [3, 5]. Importantly, HBV-HCV co-infection is associated with more severe liver disease and with a higher prevalence of liver cancer[4].

HBV-HCV co-infection involves complex viral interaction. Interference between HBV and HCV in co-infected patients resulting in the suppression of viral replication has been described [1, 5–9]. While liver disease activity and fibrosis progression are generally more severe in cases of HBV-HCV co-infection, an inverse relationship between the replication of each virus within some co-infected patients has been noted, suggesting direct or indirect viral interference [10, 11]. Challenging this notion, longitudinal studies revealed that the two viruses may replicate independently within some patients, causing fluctuations in the serum level of one virus that appear unrelated to the viremia of the other [7]. Suppression of HBV replication has been observed in patients with chronic HBV infection after acute infection with HCV; similarly, inhibition of HCV replication has been observed in patients with chronic HCV superinfected with HBV [8, 12]. In this study, we analyzed the virological features of HBV-HCV co-infected patients.

In patients with chronic HBV-HCV co-infection, more rapid hepatitis B extracellular antigen (HBeAg) seroconversion and hepatitis B virus surface antigen (HBsAg) clearance have been documented [13, 14]. Compared to HBV mono-infected patients, HBsAg carriers with concurrent HCV infection have low-level HBV viremia, low titers of HBsAg in serum, and low-levels of intracellular HBsAg [15]. Furthermore, HBsAg serum titers are significantly lower during HBV-HCV co-infection, potentially due to decreased HBV replication [16]. HCV core antigen and HCV RNA are positively correlated and appear almost simultaneously in patients’ peripheral blood, suggesting HCV core antigen may be an additional useful diagnostic marker [17–19]. However, no studies have previously correlated HCV core antigen levels with serum HCV RNA in patients with HBV-HCV co-infection. Therefore, we studied the effect of HCV replication and HCV core Ag expression in patients with HBV-HCV co-infection.

We investigated the viral interactions in HBV-HCV co-infected patients in an HCV-endemic region. This study included a large cohort of patients with chronic hepatitis due to HCV and/or HBV infections. In this study, we collected clinical and serological/virological data, compared these data among the HBV mono-infected, HCV mono-infected, and HBV-HCV co-infected groups, and correlated the findings with the degree of liver injury.

## Methods

### Study Population

A total of 3238 high risk patients had previously been enrolled in a prior HCV study entitled “Epidemiological investigation of Hepatitis C virus infection in FuYu country of Jilin Province” on September 2012 (unpublished observations).” Patients were eligible for the study if they were 18 years of age or older. Participants completed questionnaires and underwent comprehensive medical examinations including liver ultrasound, anthropometric measurements, and blood analysis to measure markers for liver function and injury, including alanine transaminase (ALT), aspartate aminotransferase (AST), alkaline phosphatase (ALP), gama-glutamyltransferase (GGT), and albumin (ALB). Additionally, two values were calculated to determine fibrosis in liver: AST to platelet ration index (APRI) and the Fibrosis-4 score (FIB-4). Other tests included assays for detecting serum HCV antibody, HBV serological markers, HCV RNA, and HBV DNA. In all test groups, individuals with hepatitis D virus (HDV) or HIV-1 infection were excluded.

Written informed consent was obtained from all individuals and the study protocol was approved by the Ethics Committee of the First Hospital of Jilin University.

### Viral Genotype, Viral Load, and Other Biochemical Measurements

Serological markers for anti-HCV, HBsAg, and anti-HIV antibodies were measured using a Thermo Scientific Multiskan Go(Kehua, Shanghai, China). HCV genotyping was performed by multicolor fluorescence PCR using an HCV RNA genotype kit (BioAssay Science & Technology Co. Ltd, China). The levels of HBsAg serological markers was measured using the Elecsys 2010 and Roche COBAS e 411 Immunoassay System (Roche Diagnostics, Grenzach, Germany). Serum HCV RNA and HBV DNA levels were measured using the COBAS AmpliPrep/COBAS TaqMan assay (Roche Molecular Diagnostics, Grenzach, Germany) with a detection limit of 15 U/mL. Biochemical assays were performed using a Synchron LX®20 Autoanalyzer (Beckman Coulter, Brea, CA). Serum HCV core Ag was measured using an Abbott Architect i2000SR Analyzer (Abbott Laboratories, Abbott Park, IL,USA) with an HCV core Ag assay kit (Abbott Japan Co., Ltd., Tokyo, Japan).

### Statistical Analysis

Normally distributed variables are presented as mean±standard deviation (SD), and differences were evaluated by Student’s t test or analysis of variance. Non-normally distributed variables are presented as median (interquartile range, IQR), and differences were tested by Wilcoxon rank-sum test or Kruskal–Wallis test, as appropriate. Categorical variables are expressed as frequencies (%), and differences were assessed using the Pearson Chi-square test. A two-tailed P value ≤0.05 was considered statistically significant. Statistical analyses were performed using the SPSS version 18.0 software (IBM/S PSS Inc., Chicago, IL).

## Results

### Characteristics of Patients

The study population included 1468 individuals with chronic HCV and/or HBV (anti-HIV and anti-HDV negative). Among them, 1200 were mono-infected with HCV (anti-HCV positive), 161 were mono-infected with HBV (HBsAg positive), and 107 were co-infected with HBV and HCV (HBsAg and anti-HCV positive). Demographic and clinical characteristics are summarized in Table 1.

**Table 1:** Demographic and clinical characteristics of the Patient cohort.

	HBV	HCV	HBV-HCV	
Characteristic	(N = 161)	(N = 1200)	(N = 107)	P Value
Age (years), mean (SD)	46.5(9.6)^▲^	54.2(8.9)*	52.1(7.3)^#^	<0.001
Age (years) <50	104(64.6)	372(31.0)	43(40.2)	<0.001
50–59	45(28.0)	511(42.6)	47(43.9)	
≥60	12(7.4)	317(26.4)	17(15.9)	
Male, n (%)	83(51.6)	710(59.2)	64(59.8)	0.175
Needle Sharing Yes	22(13.8)	517(43.4)	46(43.0)	<0.001
No	137(86.2)	675(56.6)	61(57.0)	
BMI, kg/m^2a^ (range)	24.46(17.09–38.44)	23.80(12.91–37.58)	23.88(17.63–34.48)	0.085
HCV genotype, n (%) 1b		416(49.35)	15(45.46)	
2a		283(33.57)	11(33.33)	0.740
1b/2a		13(1.54)	0(0)	
Undetermined		131(15.54)	7(21.21)	
AST, IU/L (range)	27(5–247)^▲^	35(10–494)	34(12–154) ^#^	<0.001
ALT, IU/L (range)	25(6–219)^▲^	37(4–607)	36(7–173) ^#^	<0.001
ALP, IU/L(range)	74(33–133)^▲^	77(30–750)	77(32–205) ^#^	0.003
GGT, IU/L(range)	21(9–437)^▲^	39(9–867)	41(8–539) ^#^	<0.001
TP, g/L(range)	76(55.3–122.1)^▲^	77.1(56.3–104.2)	75.3(64.6–92.8) ^#^	0.013
ALB, g/L (range)	45.7(29.5–56)^▲^	44.9(28.4–62.3)	44.5(33.4–57.2)^#^	0.005
GLB, g/L(range)	29.7(20.9–48.9)^▲^	31.75(19.3–52.5)	31.4(20.6–49.9)^#^	<0.001
TBIL, μmol/L (range)	12.95(3.4–41.5)	13.5(3–299.5)	13.25(4.2–68.5)	0.967
DBIL, μmol/L (range)	3.85(1.2–13.9)	4.3(0.3–248.4)	4.2(1.9–26.4)	0.039
IBIL, μmol/L(range)	9.3(1.4–34.4)	9.2(1.5–51.1)	9.3(2.1–42.1)	0.644
CHE, IU/L(range)	8489.5(1446–26421)^▲^	8072(546–16877) *	7239(1623–15890) ^#^	0.007
BUN, mmol/L (range)	5.24(2.53–12.39)^▲^	5.53(1.64–11.86)	5.48(2.7–9.29)	0.036
Cr, μmol/L (range)	65.2(42–108)	66.6(31–171)	65(38.1–120)	0.351
TG, mmol/L(range)	1.3(0.4–16.31)	1.3(0.34–19.43)	1.24(0.5–12.5)	0.768
TC, mmol/L (range)	4.6(2.75–8.95)	4.5(2.11–10.58)	4.44(2.8–10.4)	0.383
GLU, mmol/L (range)	4.1(2.24–13.12)	4.17(1.51–28.28)	3.98(2.69–13.31)	0.097
PLT 10^9/L(range)	202(49–336)^▲^	185.5(30–390)	180(62–377)^#^	0.041
APRI level (mean±SD)	0.2±0.24^▲^	0.34±0.44	0.3±0.34	<0.001
FIB-4 level (mean±SD)	1.58±1.14^▲^	2.39±2.15	2.3±1.88^#^	<0.001

HCV viral genotype was not available for 357 individuals in the HCV mono-infection group and 74 individuals in the HCV-HBV co-infection group; however, those with undetermined genotype results were not among the treated patients.

^▲^P<0.05, HBV versus HCV group

*P<0.05, HCV versus HBV-HCV group

^#^P<0.05, HBV versus HBV-HCV group

Quantitative variables are displayed as interquartile range (range), except age.APRI and FIB-4 level which are displayed as mean±SD BMI, body mass index; ALT, alanine aminotransferase, Normal value: 8–50.00 U/L; AST, aspartate aminotransferase, Normal value: 8–40.00 U/L; ALP, alkaline phosphatase, Normal value: 15–112.00 U/L; GGT, glutamyl transpeptidase, Normal value: 5–54.00 U/L; TP, total protein, Normal value: 60–83.00 g/l; ALB, albumin, Normal value: 35–55.00 g/l; GLB, globulin, Normal value: 20–30.00 g/L; TBIL, total bilirubin, Normal value: 6.8–30.00 μmol/L; DBIL, direct bilirubin, Normal value: 0–8.60 μmol/L; IBIL, indirect bilirubin, Normal value: 5.1–21.40 umol/L; CHE, cholinesterase, Normal value: 4300–12000.00 U/L; BUN, blood urea nitrogen, Normal value: 3.2–7.00 mmol/L; Cr, creatinine, Normal value: 44–115.00 μmol/L; TG, triglycerides, Normal value: 0.28–1.80 mmol/L; TC, total cholesterol,Normal value: 2.6–6.00 mmol/L; GLU, glucose,Normal value: 3.9–6.10 mmol/L; PLT, platelet,Normal value: 100–300.00 10^9/L; APRI, aspartate aminotransferase to platelet ratio index; FIB-4, fibrosis index based on the four factors score

Raimondo et al. previously defined the following four patterns of viral dominance in HBV-HCV co-infected patients [7]: Group (a) HCV dominance with HCV RNA >15 IU/mL and HBV DNA <2000 IU/mL; Group (b) both viruses dominant with HBV DNA >2000 IU/mL and HCV RNA >15 IU/mL; Group (c) HBV dominance with HBV DNA >2000 IU/mL and HCV RNA <15IU/mL; and Group (d) no virus dominant with HBV DNA <2000 IU/mL and HCV RNA <15 IU/mL.

The mean age of patients in HBV mono-infection (46.5 years) was lower than patients with HBV-HCV co-infection (52.1 years) and HCV mono-infection (54.2 years), but the age of patients with HCV mono-infection was higher than in patients with HBV-HCV co-infection (Table 1). No difference in the prevalence of males was observed among the different groups. However, there was significantly fewer patients who shared contaminated needles in the HBV mono-infected group (13.8%) compared to the HCV mono-infected (43.4%) and HBV-HCV co-infected (43.0%) groups (P<0.001; Table 1).

### Comparison of Virological Characteristics between Patients with HBV-HCV Co-Infection and HCV Mono-Infection

Previous reports have demonstrated that approximately 15%-45% of patients infected with HCV spontaneously clear the virus [20–24]. In this investigation, 13.1% of patients with HCV mono-infection were negative for HCV RNA, while up to 46.7% of patients with HBV-HCV co-infection had undetectable HCV RNA (P<0.001, Table 2).

**Table 2:** Comparison of serum viral Loads and HBsAg levels.

	HBV	HCV	HBV-HCV	
Variable	N = 161	N = 1200	N = 107	P Value
Serum HCV-RNA				
Positive, n (%)	none	1043(86.9)	57(53.3)	6.59E-20
Negative, n (%)	nne	157(13.1)	50(46.7)	
<4×10^5^ IU/ml	none	52243.5)	82(76.6)	5.93E-11
≥4×10^5^ IU/ml	none	678(56.5)	25(23.4)	
Serum HBV-DNA				
Positive, n (%)	152(94.4)	none	90(84.1)	2.21E-26
Negative, n (%)	9(5.6)	none	17(15.9)	
HCV load, log_10_ IU/mL(IQR)	None	5.87(3.54–6.71)	1.18(0–5.57)	1.60E-17
HBV load, log_10_ IU/mL(IQR)	3.06(2–4.28)	none	1.97(1.3–3.43)	3.68E-14
Serum HBeAg				
Positive, n (%)	35(21.7)	none	7(6.5)	1.02E-52
Negative, n (%)	126(78.3)	none	100(93.5)	
Serum HBsAg level(COI)				<0.0001
<1000, n (%)	33(20.5)	none	47(43.92)	<0.0001
1000–4000, n (%)	38(23.6)	none	8(7.48)	0.0006
>4000, n (%)	90(55.9)	none	52(48.6)	0.2408

COI, cutoff index

The median HCV RNA levels of HBV-HCV co-infected patients was significantly lower than those of mono-infected HCV patients, (1.18[Interquartile range (IQR) 0–5.57] versus 5.87[IQR, 3.54–6.71] Log_10_ IU/ml, respectively; P<0.001) as was the percentage of patients with HCV RNA level ≥4×10^5^ IU/mL (23.4% versus 56.5%, P<0.001) (Table 2).

We then repeated this analysis, excluding anti-HCV positive patients without viremia (HCV RNA negative). We found the median HCV RNA levels of co-infected patients (5.19[IQR, 1.79–6.25] Log_10_ IU/mL) were still significantly lower than those of HCV mono-infected patients (6.10[IQR, 4.95–6.79] Log_10_ IU/ml; P <0.001). We then excluded HCV core Ag negative (HCV core Ag < 3fmol/L) or anti-HCV positive patients without viremia (HCV RNA viral negative); 88 and 42 patients were included in the HCV mono-infection and co-infection groups, respectively (Table 3). Again, the mean HCV RNA levels were significantly lower in the co-infected group (5.40±1.42 Log_10_ IU/mL) than in the HCV mono-infection group (5.95±1.30 Log_10_ IU/ml; P = 0.033).

**Table 3:** Comparison of serum HCV core Ag levels and scores of the HBV-HCV co-infection and matched HCV controls.

	HCV+HBsAg	HCV		HCV+HBsAg (HCV-RNA-positive)	HCV (HCV- RNA-positive)	
Variable	N = 107	N = 107	P Value	N = 57	N = 98	P Value
Serum HCV core Ag						
Positive, n (%)	56(52.34)	78(72.90)		42(73.68)	88(89.80)	
Negative, n (%)	51(47.66)	29(27.10)	0.0019	15(26.32)	10(10.20)	0.0085
HCV core Ag level (fmol/L)(IQR)	3.14(2.09–832.0)	1830(33.26–4516)	<0.0001	661.6(2.8–5099.1)	2141.3(348.0–5039.7)	0.047
APRI level (IQR)	0.20(0.11–0.37)	0.17(0.11–0.37)	0.5443	0.21(0.14–0.34)	0.20(0.123–0.34)	0.724
FIB-4 level (IQR)	1.73(1.24–2.89)	1.61(1.13–2.27)	0.1623	1.92(1.27–2.97)	1.66(1.19–2.31)	0.327

To test whether patients with HBV-HCV co-infection have a lower level of HCV core Ag compared to patients with HCV mono-infection, we measured HCV core Ag levels in the 107 HBV-HCV patients and in 107 age- and gender-matched patients with HCV mono-infection. Similar to the HCV RNA findings, the percentage of HCV core Ag-positive patients was significantly lower in the HBV-HCV co-infected group than in the HCV mono-infected group (52.34% versus 72.90%, respectively; P<0.001) as were the mean HCV core Ag levels (3.14[IQR,2.09–832.0] versus 1830[IQR, 33.26–4516], respectively; P<0.0001) (Table 3).

There was no significant difference in the percentage of HCV core Ag-positive patients between the HBV DNA-negative/HBV-HCV co-infection group and the HBV DNA-positive/HBV-HCV co-infection group (58.82% versus 51.11%, respectively; P = 0.559) (Table 4); nor was there a significant difference in mean HCV core Ag levels between these two groups (1760±2970 versus 1434±3095 mean±SD; P = 0.509). There percentage of HCV core Ag-positive patients in the HCV RNA-negative/HBV-HCV co-infection group was significantly lower than in the HCV RNA-positive/HBV-HCV co-infection group (28.0% versus 73.68%, P<0.0001) (Table 4). There was also a significant difference in mean HCV core Ag levels between these two groups (3.576±5.906 versus 2785±3753 mean±SD; P<0.0001) (Table 4).

**Table 4:** Comparison of serum HCV core Ag levels in HBV- HCV co-infection patients.

	HBV DNA-negative	HBV DNA-positive		HCV RNA-negative	HCV RNA-positive	
Variable	N = 17	N = 90	P Value	N = 50	N = 57	P Value
Serum HCV core Ag						
Positive, n (%)	10(58.82)	46(51.11)		14(28.0)	42(73.68)	
Negative, n (%)	7(41.18)	44(48.89)	0.5593	36(72.0)	15(26.32)	<0.0001
HCV core Ag level, (fmol/L)(mean±SD)	1760±2970	1434±3095	0.509	3.576±5.906	2785±3753	<0.0001

HCV genotyping was performed in 876 HCV RNA-positive patients. HCV genotype 1b was detected in 431 patients (49.20%), genotype 2a was detected in 294 patients (33.56%), and genotype 1b/2a was detected in 13 patients (1.49%). We were unable to determine the HCV genotype in 138 patients (15.75%). The distribution of HCV genotypes did not differ between the HBV-HCV co-infected patients and the HCV mono-infected patients (P = 0.740).

The HBV-HCV co-infected group had similar ALT, AST, ALP, GGT, ALB, total platelet, APRI, and FIB-4 levels compared to those of the HCV mono-infected group (Table 1). We found no significant differences in APRI and FIB-4 levels between the 107 HBV-HCV co-infected patients and 107 matched HCV controls, or between these two groups of patients with HCV RNA viremia. (Table 3).

### Comparison of Virological Characteristics between Patients with HBV-HCV Co-Infection and HBV Mono-Infection

The HBV-HCV co-infection group had a lower percentage of HBV DNA positive patients compared to that of the HBV mono-infection group (84.1% versus 94.4%, respectively; P<0.001) (Table 2). In addition, the HBV-HCV co-infection group had significantly lower median HBV DNA levels than those of the HBV mono-infection group (1.97[IQR, 1.3–3.43] versus 3.06[IQR, 2–4.28] Log_10_ IU/mL, respectively; P<0.001). Furthermore, 6.5% (7/107) of patients with HBV-HCV co-infection had undetectable HBV DNA and HCV RNA levels. The percentage of HBeAg-positive patients in the HBV mono-infection group was 21.7% (35/161), which was significantly higher than that of the co-infection group [6.5% (7 /107); P<0.0001; Table 2].

The percentage of patients with HBsAg levels less than 1000 cutoff index in the co-infection group was significantly higher than that of the HBV mono-infection group (43.92% versus 20.5%, respectively; P<0.001, Table 2).

The HBV-HCV co-infection group had higher ALT, AST, ALP, GGT, APRI and FIB-4 levels, but lower ALB and total platelet compared to the HBV mono-infection group (Table 1).

### Baseline Characteristics of HBV and HCV Replication in Patients with HBV-HCV Co-Infection

Baseline characteristics of HBV/HCV patients are presented in Table 5. Active infection with HBV and HCV was found in 8 cases, inactive infection by both viruses in 40 cases, active HBV/inactive HCV in 20 cases, and inactive HBV/active HCV in 39 cases.

**Table 5:** Baseline characteristics of HBV/HCV replication with HBV-HCV co-infection.

	HCV+HBV(N = 107)				
	a) InactiveHBV/Active HCV	b) ActiveHBV/HCV	c) ActiveHBV/InactiveHCV	d) Inactive HBV/HCV	
Characteristic	(N = 39)	(N = 8)	(N = 20)	(N = 40)	P Value
Age (years), (mean±SD)	53.03±6.8	51.13±9.88	50.85±6.51	52.05±7.68	0.719
Male, n (%)	25(64.1)	5(62.5)	15(75)	19(47.5)	0.190
BMI, kg/m^2a^(mean±SD)	24.17±3.93	24.17±3.7	24.3±3.14	24.84±3.85	0.868
HCV RNA, log_10_ IU/mL(IQR)	6.64(3.75–6.40)	5.96(5.27–6.22)	0(0–0)	0(0–0)	<0.001
HBV RNA, log_10_ IU/mL(IQR)	1.46(0.0–1.87)	3.89(3.54–5.27)	5.46(4.09–6.07)	1.77(1.30–2.42)	<0.001
PLT 10^9/L(range)	172(90–377)	192(126–286)	163(70–261)	192.5(62–315)	0.188
AST, IU/L (range)	36(18–120)	37(24–75)	40.5(25–130)	26(12–154)	0.004
ALT, IU/L (range)	41(13–150)	36(8–145)	39.5(17–98)	20.5(7–173)	0.009
ALP, IU/L(range)	74(43–205)	76(35–79)	86.5(58–173)	78(32–149)	0.313
GGT, IU/L(range)	41(16–340)	41(14–539)	45.5(10–160)	29(8–323)	0.170
TP,g/L(range)	76.9(67.3–92.8)	74.45(68.2–79.1)	73.75(66.2–86.8)	75.3(64.6–92.4)	0.189
ALB, g/L (range)	44.3(34.8–55.4)	45.85(41.9–49)	44.9(33.4–53.3)	44.1(33.7–57.2)	0.964
GLB, g/L(range)	32.8(23.6–46.2)	28.6(26.3–33)	30(22.9–47)	30.2(20.6–49.9)	0.320
TBIL, μmol/L (range)	13.9(4.2–36.4)	12.9(7.1–38.3)	17.2(6–41.4)	11.2(5.8–68.5)	0.093
DBIL, μmol/L (range)	4.65(2.1–10.6)	3.75(2.6–8.4)	4.55(2.2–18.5)	3.6(1.9–26.4)	0.036
IBIL, μmol/L(range)	9.5(2.1–26.9)	9.15(4.3–29.9)	12.9(3.8–32.1)	7.8(3.9–42.1)	0.489
CHE, IU/L(range)	7395(1623–12263)	7473.5(5169–10112)	7190.5(2153–15890)	7288.5(1752–13390)	0.969
BUN, mmol/L (range)	5.37(2.7–9.19)	4.67(3.28–7.56)	4.99(3.48–8.74)	5.51(3.77–9.29)	0.901
Cr, μmol/L (range)	64.3(43.8–102.5)	67.9(51.1–84)	66.7(38.1–120)	65.7(43.8–106.4)	0.998
TG, mmol/L(range)	1.2(0.53–12.5)	1.24(0.69–2.6)	1.25(0.5–4.3)	1.29(0.62–7.15)	0.783
TC, mmol/L (range)	4.23(2.9–7.13)	4.52(3.61–8.78)	4.73(2.82–10.4)	4.69(2.8–6.59)	0.312
GLU, mmol/L (range)	4.22(3.01–13.31)	4.73(3.32–7.85)	3.82(2.69–7.37)	3.94(2.81–8.73)	0.364
APRI level (range)	0.23(0.07–1.29)	0.25(0.09–0.38)	0.27(0.1–1.86)	0.13(0.05–2.48)	0.014
FIB-4 level (range)	1.98(0.52–8.64)	1.63(1.09–3.02)	2.1(0.79–12.01)	1.42(0.54–11.52)	0.166
HBsAg level (IQR)	7.97(1.12–5122.0)	9235.5(6284.25–10455.5)	7441.5(6212.0–9163.0)	188.6(3.29–6207.5)	<0.001
HCV core Ag level (fmol/L) (IQR)	1726.19(25.8–66116.29)	2476.72(609.61–6546.90)	2.19(1.75–2.74)	2.24(1.82–3.41)	<0.001

Quantitative variables are displayed as interquartile range (range), and HBsAg, HCV core Ag,HCV-RNA and HBV-DNA which are displayed as interquartile range (IQR), except age and BMI are displayed as interquartile mean±SD

BMI, body mass index; ALT, alanine aminotransferase, Normal value: 8–50.00 U/L; AST, aspartate aminotransferase, Normal value: 8–40.00 U/L; ALP, alkaline phosphatase, Normal value: 15–112.00 U/L; GGT, glutamyl transpeptidase, Normal value: 5–54.00 U/L; TP, total protein, Normal value: 60–83.00 g/l; ALB, albumin, Normal value: 35–55.00 g/l; GLB, globulin, Normal value: 20–30.00 g/L; TBIL, total bilirubin, Normal value: 6.8–30.00 μmol/L; DBIL, direct bilirubin, Normal value: 0–8.60 μmol/L; IBIL, indirect bilirubin, Normal value: 5.1–21.40 umol/L; CHE, cholinesterase, Normal value: 4300–12000.00 U/L; BUN, blood urea nitrogen, Normal value: 3.2–7.00 mmol/L; Cr, creatinine, Normal value: 44–115.00 μmol/L; TG, triglycerides, Normal value: 0.28–1.80 mmol/L; TC, total cholesterol,Normal value: 2.6–6.00 mmol/L; GLU, glucose,Normal value: 3.9–6.10 mmol/L; PLT, platelet,Normal value: 100–300.00 10^9/L; APRI, aspartate aminotransferase to platelet ratio index; FIB-4, fibrosis index based on the four factors score

Because HBsAg did not correlate with HBV DNA and HCV RNA in the overall cohort, we analyzed patterns of HBV and HCV dominance in co-infected patients. Twenty patients (18.69%) showed HBV dominance (Group c) (HBV DNA >2000 IU/mL and HCV RNA <15 IU/mL). The majority of HBV-HCV co-infected patients (n = 39, 36.45%) were HCV dominant (Group a) (HBV DNA <2000 IU/mL and HCV RNA >15 IU/mL). In 8 (7.48%) patients significant replication of both viruses was detected (Group b); whereas in 40 (37.38%) patients, neither of the viruses were significantly replicating (Group d) (Table 5). The baseline characteristics of the four groups of patients did not significantly differ. Interestingly, HBsAg levels were significantly different between the groups (analysis of variance, p < 0.001). As expected, HBsAg levels were highest in patients with HBV DNA (>2000 IU/mL) (Group b: 9235.5[IQR, 6284.25–10455.5], Group c: 7441.5[IQR, 6212.0–9163.0]), lower in patients without HBV dominance (Group a: 7.97[IQR, 1.12–5122.0], Group d: 188.6[IQR, 3.29–6207.5]), and lowest in patients with HCV dominance (Table 5).

### Correlation between HCV and HBV Serum Markers

We found a significant negative correlation between HCV RNA load or HCV core Ag level with HBV DNA load (r = -0.276 and r = -0.242) (Table 6). There was a negative correlation between HBsAg levels and HCV RNA (r = -0.200, p = 0.039). As expected, high correlations were found between the HCV RNA load and HCV core Ag level (r = 0.781) as well as between the HBV DNA load and HBsAg level (r = 0.558) in patients with HBV-HCV co-infecton (Table 6).

**Table 6:** Correlation of serum markers in HBV- HCV co-infection patients (n = 107).

	HCV RNA log_10_ IU/mL	HCV core Ag level (fmol/L)	HBV DNA log_10_ IU/mL	HBsAg level (fmol/L)
HCV RNA log_10_ IU/mL	1.000	0.781**	-0.276**	-0.200*
HCV core Ag level (fmol/L)		1.000	-0.242*	-0.161
HBV DNA log_10_ IU/mL			1.000	0.558**
HBsAg level				1.000

**P<0.01, Pair-wise Spearman correlation analysis

*P<0.05, Pair-wise Spearman correlation analysis

## Discussion

Among the patients included in this study, we found that 8.4% of those with chronic HCV infection were co-infected with HBV. In China, the prevalence of HCV is approximately 0.43% in the population between the ages of 1 and 59 [25]. Therefore, there may be a large number of patients in China who are co-infected with HCV and HBV. Our study evaluated the proportion of HCV-infected patients from an HCV-endemic region in northeast China who were exposed to HBV, as well as the proportion of subjects who were co-infected with HBV.

We obtained data from a substantial number of patients, which suggests that HCV and HBV mutually inhibit the other virus’s replication in co-infected patients. Our virological data suggest that the interaction between the viruses, which ultimately determines the progression of chronic hepatitis, might be characterized by reciprocal replication inhibition of the respective viruses. However, whether the order of acquisition of virus infection plays a role in determining the degree of suppression remains to be established.

The virological patterns of HBV and HCV infections have been investigated in a large number of clinical studies [3, 26] Most of these studies were cross-sectional evaluations of HBV and HCV viral loads observed at a single time point, showing an apparent dominant role of HCV (i.e., high HCV RNA and low HBV DNA levels) in the majority of the cases. However, other reports suggest reciprocal interference or a dominant effect of HBV [5, 27–29]. Furthermore, ethnic factors that may influence the dominant role of one virus over the other have been proposed [30]; however, this phenomenon is not well understood, and the mechanisms by which this occurs have yet to be established [31, 32].

We found a marked difference in the severity of liver disease among the HCV mono-infected, HBV mono-infected, and HBV-HCV co-infected groups (Table 1). Indeed, similar differences in the clinical presentation of liver disease were reported in a recent study of 1257 patients from New York City with chronic HCV infection, including 26 patients with HBV-HCV co-infection [1]. Other studies have also confirmed that HBV-HCV co-infection is associated with more advanced liver disease compared to HBV or HCV mono-infection [5, 8, 28, 33–36].

The frequency of HBeAg negativity is significantly higher in patients with HBV-HCV co-infected patients compared to those with HBV mono-infection. It is possible that HBeAg-negative patients had seroconversion to anti-Hbe before becoming inactive carriers. Similarly, in a 6-year follow-up study, Sheen et al. found a rate of HBsAg clearance 2.5 times faster in HBsAg/anti-HCV-positive patients than in those with HBV infection alone [37]. Ohkawa et al. [30] and Pontisso et al. [29] observed a reciprocal inhibitory effect between HBV and HCV in small groups of patients. In addition, Shih et al. observed suppression of HBV expression and replication by the core protein of HCV in cell culture [38].

If HBV and HCV infect the same hepatocyte, HCV structural or functional proteins may directly influence HBV replication and HBsAg expression, or vice versa [39]. For example, HBsAg antigen expression and viremia are lower in the livers of HBV-HCV co-infected patients [16]. These findings and results from the multivariate analysis of our follow-up study suggest HCV is accountable for enhanced HBsAg seroclearance in chronic HBV infection [37].

A recent case-control study found that patients with chronic HBV infection who had serum HBsAg clearance had a significantly higher prevalence of anti-HCV antibodies than age- and sex-matched controls patients (31.4% versus 5.9%; P<0.001) [40]. This observation provides indirect evidence to support the concept that HCV superinfection exerts viral interference that can suppress or terminate a chronic HBsAg carrier state. Other recent studies have discussed indirect mechanisms that may be involved. Bellecave et al. showed in vitro that HBV and HCV can replicate in the same cell without direct viral interference [31]. Wiegand et al. found higher interferon-gamma-inducible protein-10 (IP-10) levels in HCV-dominant HBV-HCV co-infected patients, which suggests that immune mechanisms may be responsible for HCV suppressing HBV DNA replication and HBsAg production [41].

As we anticipated, there was a significant negative correlation between HCV RNA load or HCV core Ag level and HBV DNA load as well as between HBsAg levels and HCV RNA levels (Table 6). The values were similar to those reported by Wiegand et al.[41], which showed that, overall, HBsAg levels had a positive correlation with HBV DNA (r = 0.52, p < 0.001) and a negative correlation with HCV RNA levels (r = -0.20, p = 0.06). Therefore, although a significantly higher percentage of patients had a lower HBsAg level (< 1000 IU/mL) in the co-infection group compared to the HBV mono-infection group, the negative correlation between HBsAg levels and HCV RNA levels implies that the interplay between HBV and HCV may be not the result of HBsAg. It is therefore important to look for host factors that may influence the difference in viral load and serum markers observed in patients with HBV-HCV co-infection. Clearly, further research in this important area is warranted.

Our study was a cross-sectional study based on demographic and clinical data collected from a large number of samples. We did not evaluate the long-term outcomes of HBV-HCV co-infection. Because HBV-HCV viremia is a dynamic process [7], long-term follow-up will be critical to understanding the complex interactions between HBV and HCV in co-infected patients. We don't have to HCV mono-infection group (HBsAg-negative patients) with HBV DNA testing; therefore, we may missed the occult HCV co-infection with HBV (HBsAg-negative with detectable HBV DNA), and we may have underestimated the actual prevalence of HBV-HCV co-infection. HBV-HCV co-infected patients exhibited severe liver injury, which is consistent with a very high risk of liver cancer among these individuals [42, 43]. Therefore, tests should to be performed to detect for co-infected patients to proactively screen for liver cancer, and aggressive treatment may be required for these patients.

## Supporting Information

S1 Table Original data used in this study. (XLS)
